# Condensed Mitotic Chromosome Structure at Nanometer Resolution Using PALM and EGFP- Histones

**DOI:** 10.1371/journal.pone.0012768

**Published:** 2010-09-15

**Authors:** Atsushi Matsuda, Lin Shao, Jerome Boulanger, Charles Kervrann, Peter M. Carlton, Peter Kner, David Agard, John W. Sedat

**Affiliations:** 1 Department of Biochemistry and Biophysics, The Keck Center for Advanced Microscopy, University of California San Francisco, San Francisco, California, United States of America; 2 Institut Curie, Centre de Recherche, Paris, France; 3 INRIA Rennes Bretagne Atlantique, Campus Universitaire de Beaulieu, Rennes, France; 4 Howard Hughes Medical Institute and Department of Biochemistry and Biophysics, The Keck Center for Advanced Microscopy, University of California San Francisco, San Francisco, California, United States of America; Kings College London, United Kingdom

## Abstract

Photoactivated localization microscopy (PALM) and related fluorescent biological imaging methods are capable of providing very high spatial resolutions (up to 20 nm). Two major demands limit its widespread use on biological samples: requirements for photoactivatable/photoconvertible fluorescent molecules, which are sometimes difficult to incorporate, and high background signals from autofluorescence or fluorophores in adjacent focal planes in three-dimensional imaging which reduces PALM resolution significantly. We present here a high-resolution PALM method utilizing conventional EGFP as the photoconvertible fluorophore, improved algorithms to deal with high levels of biological background noise, and apply this to imaging higher order chromatin structure. We found that the emission wavelength of EGFP is efficiently converted from green to red when exposed to blue light in the presence of reduced riboflavin. The photon yield of red-converted EGFP using riboflavin is comparable to other bright photoconvertible fluorescent proteins that allow <20 nm resolution. We further found that image pre-processing using a combination of denoising and deconvolution of the raw PALM images substantially improved the spatial resolution of the reconstruction from noisy images. Performing PALM on *Drosophila* mitotic chromosomes labeled with H2AvD-EGFP, a histone H2A variant, revealed filamentous components of ∼70 nm. This is the first observation of fine chromatin filaments specific for one histone variant at a resolution approximating that of conventional electron microscope images (10–30 nm). As demonstrated by modeling and experiments on a challenging specimen, the techniques described here facilitate super-resolution fluorescent imaging with common biological samples.

## Introduction

During interphase, eukaryotic chromatin is organized into a remarkably complex and dynamic assembly of large-scale (30–300 nm) domains built from a basic nucleosomal motif [1], [2]. Changes in local chromatin condensation and organization are known to play critical roles in modulating gene expression and the maintenance of epigenetic regulation [3], [4]. As the cell cycle progresses, interphase chromatin is further condensed to become the maximally dense, structurally reproducible mitotic chromosome structure. Many biochemical studies and electron microscopy observations have suggested the existence of chromatin fibers ranging from 30 to 300 nm corresponding to intermediate folding levels [5]–[8]. However, detailed structural observations have been hindered by the extraordinary sensitivity of chromatin organization to environmental factors and the remarkably high density of mitotic chromosomes. While there have been numerous efforts to minimize these factors during EM observations [6], [9]–[13], none of the studies have fully resolved the lack of specific labeling methods or concerns of perturbations induced during the harsh sample preparation procedures required for electron microscopy. As a consequence, the existence and arrangement of such higher-order structures within the physiological cellular environment are so far unknown. Thus, developing alternative strategies for the direct high-resolution observation of chromatin structure under non-disturbing conditions and ideally with the potential for specific labeling is a high priority.

Fluorescence microscopy is a widely-used imaging method owing to its high specificity and minimal perturbation, yet its spatial resolution had been classically limited to 200–300 nm by diffraction of light. The last few years have seen the development of several “super-resolution” fluorescence imaging methods that can extend the resolution beyond the diffraction limit of light, achieving practical lateral resolutions of 20–50 nm. These include stimulated emission depletion (STED) [14], reversible saturable optical fluorescence transitions (RESOLFT) [15], saturated structured illumination microscopy (SSIM) [16], photoactivated localization microscopy (PALM) [17]–[19], stochastic optical reconstruction microscopy (STORM) [20], and its related direct STORM (dSTORM) [21], [22], and ground state depletion microcopy followed by individual molecule return (GSDIM) [23]. Particularly, localization microscopy such as PALM/STORM does not require very specialized microscope hardware and can achieve the highest practical resolution (∼20 nm) among these super-resolution methods. In localization microscopy, one collects a time series of thousands of single molecule images, exploiting stochastic activation of photoactivatable/photoconvertible fluorescent molecules to temporally separate molecules that are otherwise spatially inseparable given the size of the diffraction spots. Gaussian fitting of temporally isolated single diffraction spots can give precise localization at the nanometer level. Localization microscopy with genetically encoded fluorescent proteins makes sample preparation easier and less subject to perturbation. Its drawback, however, is the requirement for photoactivatable/photoconvertible fluorescent proteins, which sometimes demands that researchers invest significant time to recreate transgenic animals. Furthermore, low photon yield from the known monomeric photoactivatable fluorescent proteins limits the signal to noise ratio (SNR) obtainable in a biological context, often resulting in final resolutions in the range of only 50–100 nm.

Here we demonstrate that commonly used EGFP, a modified fluorescent protein from *Aequorea vi*ctoria [26], can be effectively employed as a photoconvertible fluorescent protein for PALM. We further show that a novel image processing strategy applied to the raw PALM images greatly facilitates the recovery of the peak intensity from a dim single fluorescent molecule in the presence of high background caused by either autofluorescence or out-of-focus blur, thereby leading to a more accurate determination of the centroid of each fluorophore. These improvements eliminate the barrier to photoactivatable/photoconvertible fluorescent proteins or total internal reflection (TIRF) to avoid out-of-focus blur. The techniques described here have allowed us to directly observe EGFP-labeled mitotic chromosomes at high resolution (∼30 nm) even in the presence of background in biological samples.

## Results

### Reduced flavin facilitates photoconversion of EGFP from green to red

EGFP has been known to change color under anaerobic conditions or by oxidation agents with the help of blue light [27]–[29]. The red fluorescence has a broad excitation spectrum with maxima at 500 nm in the absence of oxygen [27], or 575 nm in the presence of oxidation agents [28], and an emission maximum at 600 nm. The molecular mechanism of this red-EGFP conversion is unknown. We tried to use this EGFP photoconversion for PALM. However, the conversion efficiency was insufficient (see below for comparison of conversion efficiency), and as a result, the resolution of the reconstructed image was poor.

**Figure 1:**
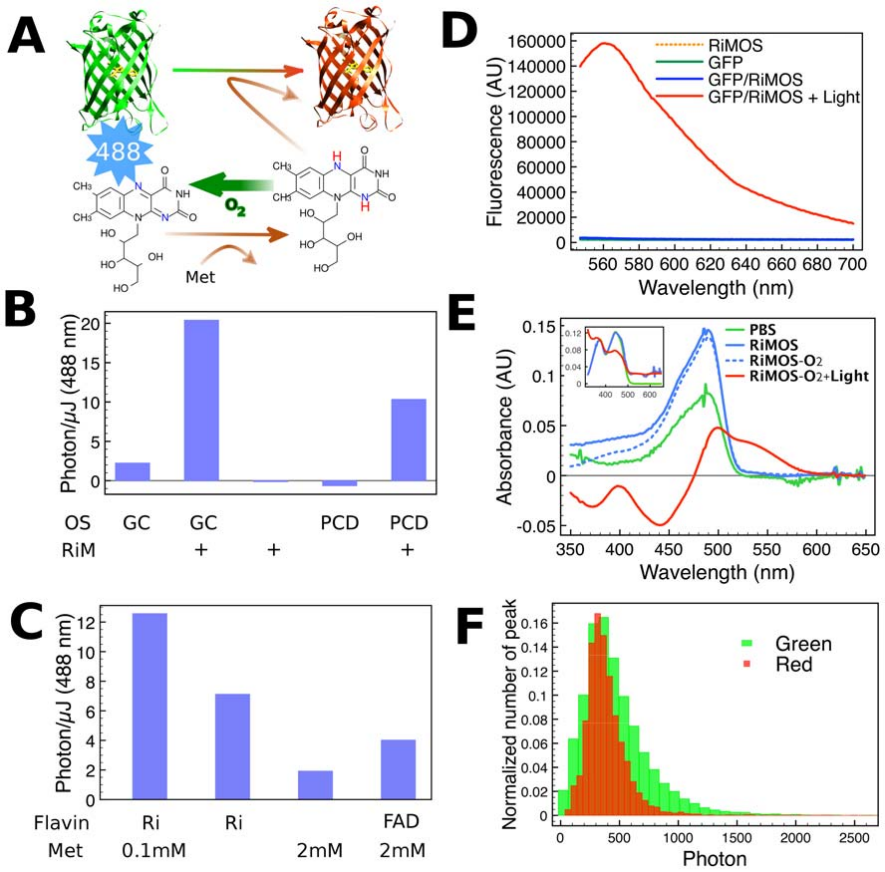
Photoconversion of EGFP by Reduced Flavin. (A) Summary of interactions among EGFP (chromophore is shown yellow), riboflavin (chemical structure), methionine (Met), molecular oxygen (O^2^), and blue light (488 nm wavelength). Photoreduction of riboflavin results in acquisition of two hydrogens (red) with a bond rearrangement between two nitrogen atoms (blue). Reduced riboflavin is easily oxidized by O^2^, but an oxygen scavenger system removes it to protect reduced state of riboflavin. (B) Photoconversion efficiency of fixed *E. coli* expressing EGFP in various surrounding media. The measurements are done as in Figure S1. OS, oxygen scavenger; GC, glucose oxidase/catalase; PCD, protocatechuate-3,4-dioxygenase; RiM, 0.1 mM riboflavin and 0.5 mM methionine. (C) Photoconversion efficiency of fixed *E. coli* expressing EGFP in the modified RiMOS (riboflavin, methionine and oxygen scavenger). Ri, 0.1 mM riboflavin; Met, methionine; FAD, 0.1 mM flavin adenine mononucleotide. Oxygen scavenger is included in all media. (D) Fluorescence emission spectra of EGFP (excitation with 532 nm). (E) Absorption spectra of EGFP in response to the change in the surrounding environment. PBS was used as blank for EGFP+PBS whereas RiMOS was used as blank for series of measurements using EGFP+RiMOS. Inset shows absorption spectra of a RiMOS solution where the color code is the same as EGFP, and PBS was used as blank (F) Histogram of number of photons emitted from EGFP in both green and red forms measured by single molecule imaging.

We noticed that flavin adenine dinonucleotide (FAD) is contained in glucose oxidase that was used to remove the oxygen in several of these reports [27], [30]. Also, reports of successful in vivo GFP photoconversion often involved mitochondrial localization where FAD is enriched [28], [30]. Flavin is reduced by blue light in the presence of a variety of amines [31], in a process called ‘photoreduction’. Its reduced state can be maintained only under anaerobic conditions. Therefore, both EGFP photoconversion and flavin photoreduction have very similar requirements for blue light and an anaerobic environment. We hypothesized that reduced flavin might facilitate changing EGFP color, and that anaerobic conditions are required to maintain the reduced state of flavin (Figure 1A). For photoconversion of EGFP, we chose riboflavin instead of FAD as it has a higher photoreduction rate [31]. Methionine is known to be a good substrate of photoreduction and does not produce formaldehyde, a byproduct common to other substrates [31]. We call the medium containing riboflavin, methionine, and an oxygen scavenger (glucose oxidase, catalase and glucose) RiMOS.

To compare the photoconversion efficiency of EGFP in various media, fixed *E. coli* cells expressing EGFP protein were immobilized on cover slips, and the increase in the red fluorescence excited by 532 or 560 nm with sparse 488-nm activation pulses was measured (see supplemental Figure S1). For quantitative comparison of photoactivation, the photon increase from the initial time point was summed over the time period and normalized by input activation laser power. Moderate photoconversion was observed in oxygen scavenger alone (GC in Figure 1B) as reported previously [27], [30]. As already mentioned above, glucose oxidase in this oxygen scavenger contains flavin as a form of FAD, which is moderately reduced by blue light in the absence of oxygen [31]. As expected from our hypothesis, EGFP in RiMOS photoconverts 9 times more efficiently compared to using the oxygen scavenger alone (Figure 1B). Riboflavin and methionine alone without the oxygen scavenger did not show any photoconversion and thus resulted only in photobleaching (causing the negative value in Figure 1B). This indicates that an anaerobic environment is required for photoconversion by riboflavin. On the other hand, another oxygen scavenger (protocatechuic acid (PCA) + protocatechuate-3,4-dioxygenase (PCD)) [32], which does not contain FAD or any flavins, did not facilitate photoconversion at all (Figure 1B), indicating that absence of oxygen alone is insufficient to support the photoconversion of EGFP. Addition of riboflavin and methionine to this PCD-based oxygen scavenger does enable EGFP photoconversion (Figure 1B). Thus while there are differences in efficiency, photoconversion does not require a specific oxygen scavenger. Photoconversion with the oxidative agent, K_3_[Fe(CN)_6_] (potassium ferricyanide) [28] was very inefficient or very dim and thus bleaching outweighed activation (Figure S1). We observed fast bleaching of green fluorescence by K_3_[Fe(CN)_6_] as reported [28] (data not shown). Bleaching kinetics of green fluorescence in RiMOS, on the other hand, was unchanged by photoconversion (data not shown).

To further confirm that flavin photoreduction is responsible for the photoconversion of EGFP, components required for photoreduction were removed from RiMOS, and the fluorescence signals from the *E. coli* sample were measured. As shown in Figure 1C, removing methionine significantly reduced photoconversion, although the final values remained high presumably owing to the ubiquitous presence of substrates for photoconversion (amine, e.g. amino acids) in this *E. coli* preparation. Removing riboflavin greatly reduced photoconversion to the basal level comparable to oxygen scavenger alone (see Figure 1B “GC”). Substituting riboflavin with FAD, which is about 10 times less efficient for photoreduction [31], also enabled photoconversion of EGFP but at much reduced rate. Thus, these data are consistent with our model that reduced flavin is responsible for changing the color of EGFP.

Next we measured fluorescent spectra before and after photoconversion *in vitro*. When purified EGFP protein solution was mixed with RiMOS, no red fluorescence was observed irrespective of the presence or absence of oxygen (Figure 1D). The red fluorescence with an emission peak at 561 nm was observed only after illumination with strong 488 nm light under anaerobic conditions (Figure 1D).

Absorption spectra were a little more complicated due to changes in riboflavin and FAD in glucose oxidase absorption. The absorption peak of oxidized riboflavin lies at 443 nm (Figure 1E, inset), irrespective of the presence or absence of oxygen scavenger. Reduction of flavins was achieved in 5 min upon illumination by strong 488 nm light under anaerobic conditions, as judged by the decrease in green fluorescence (emission maximum at 525 nm). After exposure of EGFP/RiMOS solution to 488 nm light for longer times (>15 min), a broad absorption band peaked at about 500 nm and extending to about 580 nm appeared (Figure 1E), which is distinct from the absorption characteristics of flavins (Figure 1E inset). The negative value of absorbance, around 450 nm with EGFP (red curve, Figure 1E), is thus likely due to the reduction of riboflavin and FAD by prolonged blue light illumination. In our hand, red fluorescence from EGFP was clearly observed with 532 nm laser line. Although the absorption peak for the red EGFP is around 500 nm, this wavelength also excites the green form of EGFP that exists in much larger quantity than the red form, and would make red fluorescence difficult to observe. On the other hand, although longer wavelength such as 560 nm also excites red EGFP, it excites some other components in RiMOS (possibly reduced flavin) as well, and again makes red fluorescence difficult to observe.

For PALM, the most important parameter for resolution is the number of photons emitted from a single fluorescent molecule. We measured the number of photons obtainable from the green and red forms of EGFP to examine if sufficient photons were available for high-resolution PALM. From a typical single molecule of EGFP, which is one of the brightest fluorescent proteins owing to its high photostability [33], we detected about 440 photons with our wide-field microscope before photobleaching (Figure 1F, average of 432 photons and median of 447). From a typical single molecule of red EGFP, we detected about 400 photons before photobleaching (Figure 1F, average of 414 photons and median of 382 photons).

### Improving Resolution by Image Pre-Processing

Although the average single red EGFP molecule emits sufficient photons for precise localization of <10 nm in theory [34], in practice it is often difficult to obtain such high precision in real biological samples. The main factor to reduce localization accuracy is the noise in the image. This is a universal problem inherent to all monomeric fluorescent proteins or dim organic dyes. Image noise comes from three sources: photon counting statistics, camera readout noise, and biological background noise resulting from autofluorescence and co-activated fluorophores in adjacent out-of-focus planes in three-dimensional (3D) samples. Although noise is well recognized as a dominant problem in low-photon, single-molecule studies, the importance of noise in PALM or related techniques has been understudied, and, to our knowledge, not been extensively emphasized. We present here evidence that noise can easily diminish PALM resolution much more than previously thought, but that resolution can be recovered by state-of-the-art image processing strategies.

**Figure 2:**
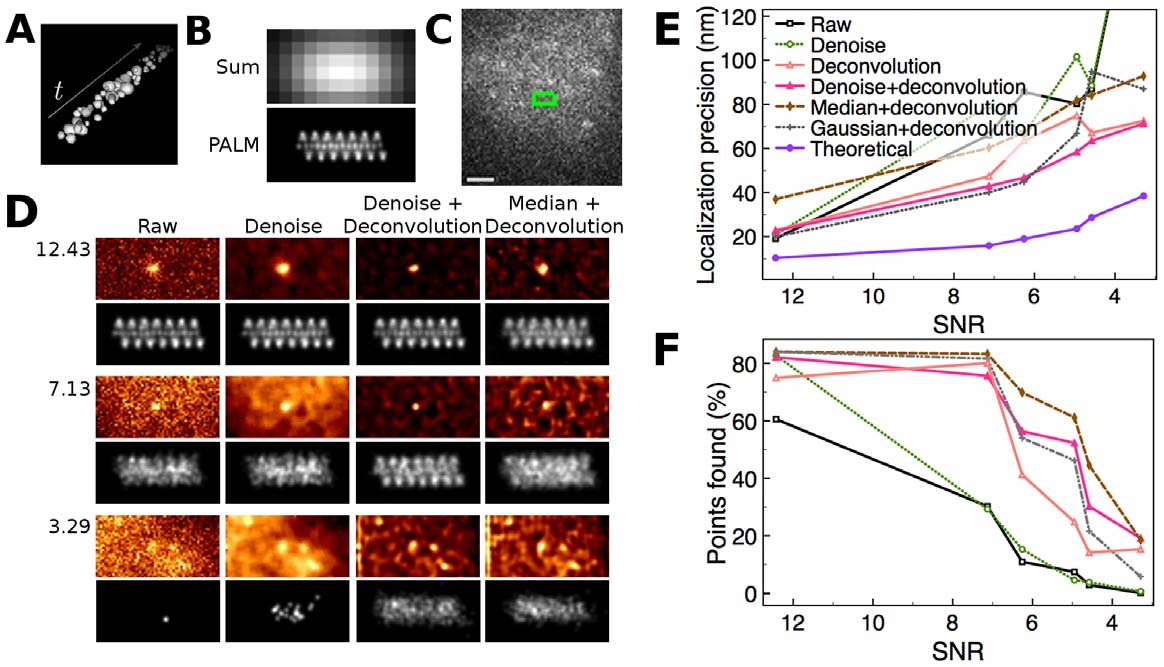
A Simulation of PALM Reconstruction in Relation to the Noise. (A) Points making up a simulated helix with a pitch of 80 nm is confined in a 5,600 nm ×160 nm ×160 nm space, and randomly scattered over 10,000 time frames. This is convolved with a real point-spread function (PSF) to imitate real microscopic diffraction spots. The central cross section was used as a raw PALM image. (B) “Sum” is the summation of all diffraction limited raw images, and “PALM” is the PALM reconstruction. (C) Acquired noise image with SD of 1.67 photons (SNR 7.13). The green box shows where the helix is to be embedded. Bar is 1.0 µm. (D) Images of different noise levels were added to the simulated raw image. Shown here are results for three noise levels corresponding to SNRs of 12.43, 7.13, and 3.29. For each noise level, shown in color are a raw image with a representative peak and the same image denoised, denoised and then deconvolved, and median-filtered and then deconvolved. Shown in grayscale are the PALM reconstructions corresponding to each of the 4 circumstances. The pixel size of the PALM reconstruction images is 1/6 of that of the raw images. (E) One-dimensional localization precision as full width of half maximum (FWHM) of the error distribution (see Fig S2A). Localization precision from the raw and the denoised images is outside of plot area at SNR 3.29 due to the unavailability of enough number of points to calculate FWHM. (F) Point finding efficiency. The graph color code in (E) applies to this plot as well.

To test only the effects of noise in PALM reconstructions, we built a simple PALM simulation using a computer-generated helix (160 nm in diameter, 80 nm in pitch) to mimic a potential mode of higher order chromatin organization. We simulated a 2D time series of raw PALM images of conventional fluorescent proteins making up the 3D helix (Figure 2A). While summing all the frames shows a diffraction-limited image with conventional optical resolution as expected (Figure 2B top), the PALM reconstruction recovers the original helix with high precision (Figure 2B bottom). The one-dimensional localization precision was 4 nm (measured by FWHM of the error distribution, see Figure S2A) and point finding efficiency was 96%. This localization precision was similar to 9 nm calculated based on the previously reported mathematical theory [34]. A series of real biological background images with different levels of background noise taken by our microscope was then added to this artificial time series (image with mid level of noise is shown in Figure 2C). PALM reconstructions at several different noise levels revealed that images with only very low noise (mean number of photons (MNP) 1.71 or with very high signal-to-noise-ratios (SNR) 12.43) permitted acceptable PALM reconstructions (resolution 20 nm, Figure 2D). This noise level was almost exclusively from camera readout noise. Slight increases in the noise level (MNP 5.55 or SNR 7.13) totally demolished PALM reconstruction (Figure 2D, E, “Raw”), even though the theory predicts high localization precision of 16 nm (Figure 2E). With our microscope and samples, we often encountered noise levels comparable to this mid level of noise (Figure 2C), and they are clearly too high for PALM reconstruction. As shown in Figure 2E, commonly used mathematical resolution approximations of least-squares Gaussian fitting of the PSF [34] deviates more with increasing noise (compare “Raw” and “Theoretical” in Figure 2E). This demonstrates that noise is a significant problem in PALM.

A patch-based, adaptive denoising algorithm [35], which was recently successfully applied to improve our live cell imaging [36], was applied to PALM raw images (Figure 2D, “Denoise”). As expected, it reduced the destructive effect of noise without reducing resolution. However, this processing alone did not help to improve resolution of PALM reconstruction (Figure 2D).

Localization precision depends mainly on the number of photons, the noise, and the width of the photon distribution (the point-spread function, PSF), often treated as a Gaussian peak. To improve resolution, we applied constrained iterative 2D-deconvolution [37] to sharpen the Gaussian peaks of the denoised image. After deconvolution, Gaussian peaks stood out more clearly and sharply as expected (Figure 2D “Denoise + Deconvolution”). Deconvolution also flattened contours caused by uneven background intensity while preserving PSF-like objects. This helped to find more points whose peak intensities were otherwise below a practical threshold (Figure 2F). A helical shape is clearly seen in reconstructions from the low and mid noise levels with a precision of 23 and 43 nm, respectively. A clear cylindrical shape is still recognizable even in reconstructions from the highest noise level (Figure 2D). These results demonstrate that the deleterious effects of realistic noise levels can be overcome by the use of advanced image processing of the raw images.

With iterative deconvolution alone (with relatively low wiener value with periodical softening), it was still able to get resolution improvement (Figure 2E, F). However, the effect is moderate and not consistent among SNR compared to the case after denoising (Figure 2E, F). Sometimes we had greater number of false positives due to high levels of noise (data now shown). Thus, although deconvolution was the key pre-processing step for resolution improvement, low-pass filtration before deconvolution is preferred to limit the buildup of high frequency noise during the deconvolution process [37]. In order to see whether alternatives to the patch-based denoising algorithm could also be used before deconvolution, we tested several more facile low-pass filters. Median filtering works well for the mid-level noise images as judged by visual perception (Figure 2D, “Median+Deconvolution”) and by the improvements in point finding efficiency (Figure 2F). However, PALM reconstruction from the median filtered images did not show as clear a periodicity as the reconstruction derived from denoised raw images, indicating that denoising provides superior localization precision. A few iterations with a Gaussian filter with small sigma value (0.79 µm) provided results similar to denoising at SNR levels higher than 6.26 in our simulations (Figure 2E, F). Thus this filter could be used as an alternative to denoising in samples with medium to high SNR.

### Metaphase Chromosomes Consist of Fine Fibers

**Figure 3:**
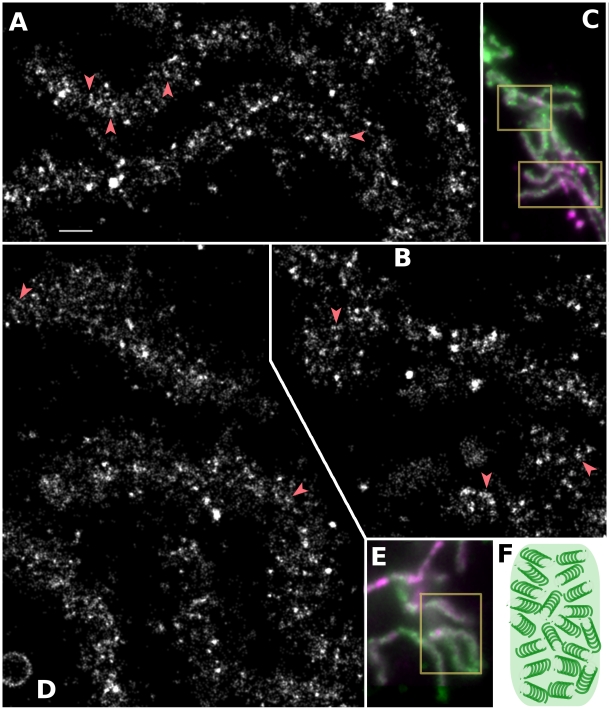
*Drosophila* Metaphase Chromosome Arms Consist of Fine Chromatin Fibers. (A, B) PALM reconstruction of chromosome arms boxed in (C) free from cytoplasm. (D) PALM reconstruction of chromosome arms boxed in (E) surrounded by the cytoplasm. Red arrowheads point to typical filamentous structures, although this structure is almost everywhere in the chromosome arms. (C, E) Denoised wide-field images of chromosomes. DAPI staining is shown in purple and H2AvD-EGFP is shown in green. Bar shown in (A) corresponds to 0.5 µm in (A, B, D) and 3 µm in (C, E). (F) A schematic drawing of the metaphase chromosome arm based on the interpretation of the PALM reconstruction.

Our PALM method using EGFP and image denoising/deconvolution was applied to the direct visualization of higher order chromatin structure within *Drosophila* prometaphase/metaphase chromosomes. The *Drosophila* embryo is an excellent source of mitotic chromosomes due to its rapid, synchronized nuclear divisions without cytokinesis that result in >5,000 nuclei in a shared cytoplasm [38]. An EGFP fusion of histone H2AvD was used for imaging. H2AvD is encoded by a single copy gene homologous to histone H2A variant H2A.Z, and accounts for 10% of total H2A histone [39]. It is widely distributed in the genome, though often enriched in gene regions irrespective of transcriptional activity and found less in heterochromatin [40]. *Drosophila* embryos were staged by *in vivo* observation at low magnification. To image mitotic chromosomes with low background fluorescence, staged *Drosophila* embryos were gently punctured and mixed with a fixation buffer. In this way, chemical fixation of chromosomes works very rapidly and many chromosomes are close to the cover slip, some of which are free from the surrounding cytoplasm. A single optical section of conventional wide-field imaging was used instead of TIRF microscopy since the chromosome thickness (500 nm) is larger than the depth of the evanescence field around the cover slip (∼100 nm). Although the exact resolution of our PALM is hard to estimate after denoising and deconvolution, the simulation results (Figure 2) suggest that the localization precision should fall within the range of 20–40 nm. Another resolution limit for PALM is the Nyquist sampling rate determined by point finding efficiency [41]. Owing to the very high photoconversion efficiency of EGFP with RiMOS (Figure 1B) and efficient deconvolution after denoising (Figure 2F), the resolution limitation by Nyquist sampling rate (∼8 nm) was much lower than the localization precision, even after our reconstruction program summed up points with short blinking into a single point (see Materials and Methods for reconstruction program). PALM reconstructions of prometaphase/metaphase chromosomes show fine structural details never observed before by conventional fluorescence microscopy (Figures 3A, D). In the PALM reconstructions (Figures 3A, B, D), chromosome matrix looks sparse most likely due to the limited localization of this histone variant in the genome. However even in the regions enriched with this histone variant, chromosome arms appear to be decomposed into numerous filamentous blocks having a cross-sectional diameter of ∼70 nm (ranging from 40 to 90 nm, Figure 3, red arrowheads). The filament-like structures were not continuous throughout chromosome arms in these two-dimensional images.

A careful examination of these 70-nm fibrous structures revealed that, although obscure in every case, they were composed of even finer stripes of ∼35 nm each separated by about 30 nm. Exact dimensions are hard to know for it is very close to the resolution limit expected for the reconstructed image of this sample. This pattern is unlikely to be a reconstruction artifact, since such clear stripes were not observed outside of the chromatin region of the slide (Fig S3), the stripe angles were variable, and such stripes were not observed in any of the simulations (Figure 2).

In addition to chromosomes fixed in the buffer (Figure 3A, B, C), we examined the structure of fixed chromosomes surrounded by the cytoplasm (Figure 3D, E) or in another fixation buffer having a different chemical composition (Figure S4, see Materials and Methods for the composition of fixation buffers). In both cases the same structural features were observed. These results suggest that chemical fixation worked very rapidly in this sample preparation, and that the chromatin structure observed is unlikely to be an artifact induced by a particular surrounding environment.

The PALM reconstruction of the data set shown in Figure 3 without using denoising and deconvolution is shown in Figure S5, where structural features are not as obvious as in Figure 3. This demonstrates again that denoising and deconvolution are important pre-processing for PALM resolution.

## Discussion

### Reduced Flavin Turns Green EGFP Red

First discovered in 1997 [27], it has been known that anaerobic conditions induce photoconversion of GFP. The reaction was suggested to require two steps due to its slow brightening (exponential time constant of about 0.7s) and that photoconversion occurs in numerous variants of GFP [27]. The fluorescent lifetime of red GFP was measured as 2.11 ns [30], close to the green form (2.4 ns). However, there had been some confusion regarding GFP photoconversion (such as [42]) possibly because the photoconversion was not readily observed under conventional anaerobic conditions alone (without glucose oxidase, see Figure 1). Our finding that anaerobic conditions are required only for maintaining a reduced flavin state explains the previous inconsistent observations. Anaerobic conditions are not directly required for GFP photoconversion, but are required to slow re-oxidization of flavin, which quickly proceeds in the presence of molecular oxygen. Also the photoconversion reaction presumably consists of at least two chemical reactions: the photoreduction of flavin and the reaction of reduced flavin with GFP of unknown mechanism. Since reduction of flavin occurs by a light-mediated process, photoconversion of GFP can be controlled by light in much the same way as other photoconvertible fluorescent proteins.

Photoreduction of flavin with thiol (methionine) produces byproducts including radicals. It is unlikely, however, that any byproducts are responsible for photoconversion of EGFP according to the results presented in this report. Photoreduction process should occur irrespective of the presence or absence of molecular oxygen, and thus byproducts should be produced also in the absence of oxygen scavengers. As seen in Figure 1B, no photoconversion was observed in the absence of oxygen scavengers. This observation strongly argues against the idea that byproducts of photoreduction are the responsible factors for photoconversion. This same observation similarly disagrees with the idea that EGFP was oxidized by the photoreduction process of flavin, since if such oxidative photoconversion like those with K_3_[Fe(CN)_6_] is in fact the chemical basis, then it should occur in the absence of oxygen scavenger. Requirement for methionine for efficient photoconversion (Figure 1C) also support this conclusion. Therefore, our data clearly point to the requirement of the reduced flavin for efficient photoconversion of EGFP.

We do not know the chemical basis by which reduced flavin changes the color of EGFP. Violet light is more efficient than blue light at photoconversion of wild-type GFP [29], which has an absorption peak at the violet region, suggesting that, in addition to photoactivating flavin, GFP must use light energy to change its color. Of particular interest is the red chromophore in the red EGFP and the role of reduced flavin. The red fluorescence of EGFP lasted hours after blue light illumination under anaerobic conditions as reported previously ([27], [30] and this report), whereas reduced riboflavin oxidizes under the same anaerobic conditions within 20 min (measured by reappearance of green fluorescence from riboflavin). This suggests that reduced flavin is required only for the initial photoconversion of EGFP and not to maintain the converted state. Diverse emission maxima have been reported depending on the photoconversion method ([27], [28] and this report) suggesting the existence of multiple red states in EGFP. In our method using riboflavin, we do not know if the EGFP chromophore itself has changed or if it uses flavin as a second chromophore. Flavin semiquinone radical, which has an absorption in the 500–650 nm range, could be produced by the photoreduction process of flavins [43], [44]. Flavin semiquinone is particularly stable in glucose oxidase [45]. A consistently higher photoconversion rate in the presence of glucose oxidase as the oxygen scavenger compared to PCD-based oxygen scavenger was observed (Figure 1). Further work is necessary to clarify the red chromophore and how GFP changes its color.

### Use of efficient photoconversion of EGFP in biological imaging

We showed that red EGFP was sufficiently bright for conducting superresolution imaging using PALM/STORM methodology. Although direct comparison is difficult, the number of photons detected from red EGFP in our microscope setting is similar to that from Eos2 (360 photons) [46], the brightest monomeric photoconvertible fluorescent protein so far reported. It is worth noting that the photoconversion of EGFP was accomplished by supplying cofactors in the surrounding media. This approach to exploit existing EGFP fusion proteins for super resolution microscopy should facilitate research in many model systems where making transgenic animals takes a significant time. Even in model systems where making transgenic strains is simple and fast, our technique would allow a large-scale PALM analysis on existing EGFP-fusion gene library. All existing EGFP-fusion genes, of which thousands are already available in laboratories and public depositories (including nearly the entire yeast proteome [47]), can be immediately utilized for high-resolution imaging with this technique. Making use of common fluorophore for superresolution microscopy has been already realized using organic dyes and fluorescent proteins [21]–[23]. Genetically encoded probes are usually preferred due to availability of higher labeling density than antibody-based staining, and allows more facile sample preparation like the chromosome preparation method employed in this study. Our EGFP-based PALM approach allows easy but high quality super-resolution imaging.

Photoconversion by cofactors described here still remains challenging for most live cell experiments due to requirements for (i) delivery of non-membrane-permeable riboflavin into the cell and (ii) the necessity of anaerobic conditions. It may be possible in the future to overcome these requirements for live cell imaging by better understanding the photoconversion chemistry. Also, even in a fixed sample, penetration of riboflavin into the biological macromolecular complex or cell membrane/wall is another potential concern, even though the molecular weight of riboflavin is very small. For example, cell wall needs to be cleaved when applying RiMOS for fixed yeast cells (unpublished results).

### Restoration of Noisy PALM Images by Image Pre-Processing

We conducted previously difficult PALM imaging with the relatively small photon budget available from fluorescent proteins in chromatin structure, a relatively large biological sample (about 500 nm in thickness) that requires wide-field, rather than TIRF, imaging. We showed the first extensive analysis of the effects of noise on PALM resolution using localization precision and Nyquist sampling rates as metrics. Surprisingly, only a slight increase in noise substantially diminished obtainable PALM resolution in our simulations (Figure 2). This raises a question about the reliability of the commonly used mathematical formula for estimation of localization precision. This discrepancy may come from different noise models. We used a complex biological image taken by our microscope whereas the mathematical theory of Thompson et al. [34] assumed a simple noise level, *b,* “is constant across the region of the spot being localized”. Based on our simulations a better estimate can be obtained by replacing the *b^2^* in the one-dimensional resolution formula (Equation 14 in [34]) by *b^3.5^* using raw data, and *b^3^* with deconvolved data. This is purely an empirical modification to the formula without any theoretical reasons for choosing these exponents. Recent reports suggests even different resolution estimates for localization microscopy [48], [49]. It is likely that more work is necessary to estimate resolution in the presence of noise in biological imaging.

Despite the noise problems, we showed for the first time that image filtering and deconvolution could improve the localization accuracy of Gaussian peaks, particularly in noisy images. Minor drawbacks such as additional processing time (Figure S2D) may be resolved by further optimization of the code.

The quantitative analysis of the effect of noise in localization precision of a point object and the benefits of digital image pre-processing presented here is applicable to single molecule and live cell imaging [36], and even fields outside of biological imaging.

### Filament-like Structures in *Drosophila* Metaphase Chromosomes

We present here the structure of metaphase chromosomes labeled with one histone variant H2AvD at a resolution of about 30 nm (see Materials and Methods). Although mitotic chromosomes are otherwise hard to image by high-resolution methods, our sample preparation for mitotic chromosomes using *Drosophila* syncytial blastoderm embryos eliminated massive cellular background, and enabled efficient sample flattening and rapid chemical fixation. The chromosome structure, however, must be viewed with caution since these chromosomes may be subject to some mechanical forces when they come out of the living embryo before chemical fixation has taken place. The chemical fixation with formaldehyde itself could be another concern to disturb microstructures. Also our PALM image came from one 2D plane with only about 200 nm focal depth above and below the true focus (data not shown, see Materials and Methods). Thus our PALM image of mitotic chromosomes may represent only a subvolume of the real structure *in vivo*. Nevertheless our structural observations are worthy of some discussion in relation to previous reports.

Our interpretation of the metaphase chromosome structure is drawn (Figure 3F) as an assembly of 70-nm filamentous blocks composed of stripes of 35-nm sub-filaments having a pitch of 65 nm, blank cavities, and seemingly unstructured chromatin matrix. The 70 nm filaments described here could be the same structure as the 60–80 nm fibers observed in mammalian interphase nuclei, or the 100–130 nm fibers in early G1 or prophase observed using electron microscopy [5], [7]. Very fine stripes <35 nm in width and 65 nm in pitch are observed as substructures within the 70 nm filaments. It is tempting to assume that these stripes are the so-called 30 nm filaments commonly observed *in vitro*, but as yet not observed *in vivo* [50]. However, confirmation will require even higher resolution than the present study.

It has been suggested that there is a non-histone core running across the long axis of the chromosomes [51], [52]. Such axis was unrecognizable in images of our PALM reconstruction, probably due to non-ubiquitous distribution of the histone variant used as a probe. Alternatively, since many observations on axial proteins have come from diffraction limited fluorescence microscopy, the core width may have been exaggerated. It could actually be very fine and/or consist of multiple lines (also see [53]). Thus if the core's diameter is smaller than our effective focal depth (400 nm), then its visibility could be obscured by points above and below it. 3D PALM imaging could resolve this problem.

An important highlight of this paper is that PALM technology is an ideal tool to dissect complex cellular structures with metaphase chromosomes being a classical example. Our PALM technology using existing EGFP-fusion genes and image pre-processing quickly achieves a 30–40 nm resolution level with all the specificity of a fluorescent protein label, a result that is impossible or at least difficult to achieve by other current technologies such as EM tomography. Furthermore, numerous EGFP-fusion constructs encoding interesting nuclear proteins are immediately available for super-resolution microscopy. It may well be the technique of choice to solve the longstanding chromosome structure problem. The remaining challenges in three dimensions are also within reach [54]–[57].

## Materials and Methods

An ethics statement is not required for this work.

### Sample Preparation for Fluorescent Proteins

Our custom bacterial expression vector pETBio was made by inserting a synthetic oligonucleotide encoding a biotinylation peptide (GLNDIFEAQKIEWHED, [58]) followed by a flexible linker with a multicloning site (*Eco*RI, *Kpn*I, *Age*I and *Bam*HI) into the *Nde*I-*Sac*I site of pET28a (Novagen). A PCR fragment containing the full coding region of EGFP (S65T and a neutral mutation of Q80R) amplified from pC4BAvDGFP [59] was inserted into the *Kpn*I-*Bam*HI site of pETBio and nucleotide sequence was confirmed by sequencing the insert. The resulting fluorescent proteins, when expressed in *E. coli* strain BL21(DE3) (Invitrogen) cotransformed with pACYC-184 (AVIDITY), which has the biotin ligase *birA* gene, along with 10 mM biotin for biotinylation, protein would have a 6x His tag at the N-terminus followed by the biotinylated peptide and fluorescent protein. The fluorescent proteins in 10 ml *E. coli* grown in LB medium were solubilized by BugBaster (Novagen) and purified by Talon column (Clontech). The protein solution was dialyzed against PBS and insoluble protein was removed by brief centrifugation. The typical yield of fluorescent proteins was 0.4 mg.

### Solution for Photoconversion

The components of RiMOS are 0.01 mM riboflavin (BioRad), 1 mM DL-methionine (Sigma), 0.5 mg/ml glucose oxidase (Sigma), 40 µg/ml catalase (Sigma), 5% glucose, 60 mM PIPES (pH 7.0). Riboflavin stock solution consisted of 0.1 mM riboflavin and 0.02 N HCl and can be stored at 4°C for 6 months. Methionine was prepared as 100 mM concentration in water freshly from powder every time. Glucose oxidase was prepared as 10 mg/ml stock solution in phosphate-buffered saline (PBS) and stored at 4°C for a few months. Catalase was prepared as a 2 mg/ml stock in PBS and stored at 4°C for 2 weeks. Glucose was prepared as 10% filter-sterilized stock solution and stored at 4°C. PIPES was prepared as 1 M stock (pH adjusted by NaOH) and used to compensate pH change by gluconic acid produced by glucose oxidase activity. RiMOS should be combined with an appropriate salt/buffer such as PBS at 1X final concentration.

### Photochemical Spectra

EGFP solution was diluted to 30 µM or 1 µM for absorbance or fluorescent spectrum measurements, respectively, in PBS or PBS/RiMOS. Absorbance was measured with a 8453 UV-Visible spectrophotometer (Agilent), normalized with either PBS or PBS/RiMOS. Excitation/emission spectra were measured with Fluoromax-3 spectrofluorometer (HORIBA) at 25°C. To make an oxygen-free environment, a layer of light mineral oil was put on top of the protein solution containing RiMOS and incubated for 1 hr at room temperature. For photoconversion, cuvettes were put behind a collimator (which broadened the light to about the size of the cuvet) on the light path of the microscope laser (488 nm, ∼80 mW) for 30–60 min at 23°C.

### EGFP Imaging Sample Preparation

For *E. coli* imaging, a solution of *E. coli* expressing fluorescent protein was incubated on the poly-L-lysine coated cover slip (Gold seal, No. 1.5) for 15–30 min at room temperature, then washed with mounting media. For single molecule imaging, cover slips were cleaned with a plasma cleaner (Solaris Model 950, Gatan) with an H_2_/O_2_mixture for 2 min, then coated with poly-L-lysine. EGFP protein (10 ng/ml in PBS) was absorbed onto the surface for 30 min at room temperature. The cover slips were extensively washed with PBS, and mounted with PBS or PBS/RiMOS. The cover slips were sealed with rubber cement.

### Chromosome Sample Preparation for PALM

As a phyisological buffer to protect chromosome integrity, Buffer A (15 mM PIPES (pH 7.0), 80 mM KCl, 20 mM NaCl, 2 mM EDTA, 0.5 mM EGTA, 0.5 mM spermidine, 0.2 mM spermine, 15 mM beta-mercaptoethanol), which was designed to protect polytene chromosome structure [6], was used as the primary buffer. In some experiments, instead of buffer A, PBS(+) was used, which is a variant of PBS supplemented with 0.9 mM CaCl_2_and 0.33 mM MgCl_2_. The fixation buffer consists of 2–3% formaldehyde, 1 µg/ml DAPI and Buffer A or PBS(+). The mounting medium consists of 15% glycerol, RiMOS and Buffer A or PBS(+). Cover slips (Gold seal, No. 1.5) were cleaned with a plasma cleaner (Gatan) with H_2_/O_2_for 2 min, then coated with poly-L-lysine. Fresh fly embryos collected from H2AvD-EGFP expressing fly [59] were aged 1.5 hr at room temperature. Then embryos were dechorionated by hand, put on a 1–2% agarose gel with 0.1 mM EDTA (as preservative) and the nuclear division cycle was followed by our low magnification fluorescent microscope “LMX” [36]. On LMX, EGFP fluorescence was observed by Hg lamp using FITC filter set, with the illumination intensity reduced to 25% by a diaphragm. Syncytial blastoderm embryos at mitotic cycle 12 or 13 were picked up, put on the cover slip close to a drop of the fixation buffer (0.5 µl) on the center of the cover slip, and the embryo was punctured by forceps and momentarily mixed with the fixation buffer. This is incubated for 5–15 min at 23°C in a moist chamber, then 4.5 µl of mounting medium was added, mounted on a glass slide, and sealed with rubber cement. The slide was incubated at room temperature for 1 hr to enzymatically remove oxygen.

### PALM Image Acquisition

The PALM images were taken by our custom wide-field microscope platform, “OMX” [36], [60]. The objective lenses used were UPlan-SApo 100x PSF NA 1.40 oil-immersion (Olympus) and UApo 150x TIRF NA 1.45 oil-immersion (Olympus), corresponding to the CCD pixel size of 0.0792 and 0.0528 µm, respectively. Type DF immersion oil of refractive index 1.515 (Cargille) was used. Red fluorescence from red EGFP was excited by a 532 nm laser (36.5 W/mm^2^) or a 560 nm laser (20.0 W/mm^2^). Exposure times were 30 and 50 ms for 100x and 150x objectives, respectively. The cameras used were back-thinned EMCCD cameras (Andor iXon897) with EM gain setting 220 at a readout speed of 10 MHz. Photoconversion was induced by short pulse (10–50 ms) of 488 nm laser (21.3 W/mm^2^) once every ten time frames. When many EGFP molecules were available, exposure to excitation light of 532 nm alone could photoconvert enough population of EGFP into red (Figure S1C). Often even with 532 nm illumination, photoconversion was too much and therefore the first 6,000–10,000 time frames were used only to bleach some EGFP population and its image was not used for reconstruction. Increasing shutter closure time between each 532 nm excitation also helped to reduce unfavorable photoconversion. For correction of stage drift, chromosome images stained with DAPI were taken once every 200 time frames with a 405 nm laser (1.34 W/mm^2^) with 10 ms exposure. The images were used afterwards for drift correction during PALM reconstruction. A typical PALM raw image consists of 15,000–25,000 time frames of 256×512 pixel images.

### PALM Image Pre-Processing

A small region of interest (typically about 200×200 pixels) of a raw image was cut out, then processed with 2D (xy) or 3D (xyt) denoising [35]. The denoised images were then deconvolved with a point spread function (PSF) carefully averaged over 30 bead images prepared for each objective, with a very small vertical axis increment of 20–40 nm to find the exact focal plane more accurately. The averaging was done with our custom Python program, which takes into account the fact that the lateral center of the PSF may change along with the vertical axis. Constrained iterative deconvolution was performed as described [61] using the enhanced-ratio method with a Wiener value of 0.8 for 30 iterations. Drift correction images were also subjected to 2D denoising.

### PALM Reconstruction

PALM reconstruction of denoised and deconvolved images was done with our custom software written in Python. The program consists of six steps.

The first step is automatic thresholding and least-square fitting of local maxima with an elliptical Gaussian function to deduce the peak intensity, position, width in two dimensions, and the background intensity.

The second step rejects peaks that are too dim, too wide, too sharp or too skewed. Here the correct cutoff value is difficult to know beforehand, since denoising and deconvolution tend to modify original peak shapes. Therefore the program adaptively finds the cut off value from the distribution of the main population (Figure S6). Peaks with irregular shapes were thrown away. This cleans up false positives from the final reconstructed image with improved resolution (Figure S6C).

The third step estimates and corrects for the stage drift. A correction image (usually a DAPI image) was processed sequentially with denoising, a Mexican-hat filter, and a Wiener-type high-pass filter using a Gaussian distribution as a contrast transfer function then cross-correlated with a similarly treated reference image. The lateral movement was estimated by Gaussian fitting of the peak of the cross-correlated images and the movement over time was smoothened with a low-pass Gaussian filter. Drift between successive PALM raw images is then interpolated from this movement estimated from the correction images. Finally, the coordinates of peaks were shifted by these calculated drift values.

The fourth step is grouping and summation of identical molecules at the same position through time. The allowable distance for identification of peaks as the same molecules was 40 nm. The program also takes into account the fact that single molecules tend to blink in fluorescence intensity, and thus a fluorescent peak reappeared after a short dark state (shorter than 50 ms in the present case) can still be regarded as the same molecule. Images of fluorescent peaks spanning multiple time frames are translated to compensate stage drift, summed up and each peak was again fitted to a Gaussian function. Rejection of peaks was repeated with cut off values determined in step 2.

The fifth step converts peak attributes to meaningful values, such as intensity to number of photons (peak and background), pixel to µm. Again, peaks with too dim or too high backgrounds were rejected based on the distribution.

The sixth step reconstructs a high-resolution image from the peak information. Each peak was rendered as a Gaussian peak with constant full width at half maximum (FWHM) value (25 nm in the case of Figure 3). The image was zoomed up by a factor to satisfy the Nyquist-Shannon sampling theorem. Thus for FWHM of 25 nm, the final image was zoomed up to get a pixel size smaller than 12.5 nm.

PALM reconstruction data were analyzed by our custom program associated with reconstruction software. Resolution limit by point density in reconstructed PALM images was calculated by taking the median of distances between nearest-neighbor points of each point in the reconstruction image and multiplying this value by two according to the Nyquist-Shannon sampling theorem.

The resolution is also limited by localization precision of each point. This resolution limit was estimated from Figure 2E using signal to noise ratio. For example, for the calculation of the final resolution, if resolution limit by sampling rate was 8 nm, while resolution limit by localization precision was 30 nm, then 30 nm was the overall resolution in our criteria.

### PALM Simulation

A 3D helix of 554.4 nm ×158.4 nm ×160.0 nm in xyz coordinates with a pitch of 79.2 nm and line width of 23.8 nm in 3D contained one point in every 7.92 nm in X dimension. Each point was rendered in a 3D Gaussian sphere with a FWHM of 79.2 nm. To make the simulated PALM images, a total of 3990 points were spread randomly over 10,000 time frames (Figure 2A). The mean number of photons recorded from single spots was set to about 250 with a normal distribution of variance of 50 photons. The mean lifetime of individual molecules was 40 ms (1.33 frame) with exponential distribution (lambda  = 0.7), which was determined from real recordings of red EGFP single molecules (data not shown). Some points show blinking just like red EGFP proteins (exponential distribution of lambda 1.3, data not shown). This image was convolved with a real 3D point spread function (PSF) to make realistic diffraction spots. The PSF used for the convolution was obtained by averaging 30 3D PSFs acquired with our 100x objective (NA 1.40) and red fluorescent beads of 50-nm diameter (Molecular Probes) excited with a 532 nm laser. A 2D slice through the middle of the helix was used as raw PALM image.

Noise images derived from actual PALM images of *Drosophila* chromosomes used the same lens and same excitation wavelength as PSF, but most EGFP molecules had been already photobleached. SNR was calculated by a mean peak height of PSF (11.15 photons) divided by the standard deviation of noise (in photon).

For pre-processing raw PALM simulation images, we varied the denoising algorithm's parameters such as adaptivity [35] (0–2), number of iterations (4–6), and dimensionality (2 or 3), and they all gave almost identical results in terms of localization precision and point finding efficiency. Therefore, only the result using adaptivity 0, 4 iterations, and 2D processing was shown. Median filter in “2D filter” program of the Priism suite (http://www.msg.ucsf.edu/IVE/) was performed with 1 iteration with 3×3 kernel size. Gaussian filter in Priism suite's “2D filter” was done with 5 iterations with 3×3 kernel size and a sigma size of 1.0 pixels. Iterations with a small Gaussian like this remove noise with minimum blurring effect unlike single convolution with a larger Gaussian. Deconvolution parameters were the same as above.

PALM reconstruction from the simulated data did not use screening for photon number and noise level. Number of photon in the simulation was not as variable as in the real fluorophore (Figure 1F), even though we tried to mimic the real distribution. Various thresholds for point finding were tried and only the best result was shown. From these PALM results, the known and localized coordinates were compared and taken as the same spots if they were in the corresponding time frame and closer than a certain distance in 2D space. We used an approximated distance calculated by (1.22lambda)/(2NA) and divided by 2 (we only need radius). The resulting distance of 125 nm would be small enough to find the corresponding peak in the simulated time series since there is usually only one peak in this distance in a single time frame. If more than two points were found within 125 nm space, then closest point was chosen. One-dimensional error in localization precision was calculated by taking the average of each difference between known and localized coordinates in X and Y dimensions. One-dimensional localization precision was then calculated as the FWHM of the Gaussian fitting of the error distribution histogram, and then multiplied by 2√2ln2. The center of the histogram in the X direction was often not at zero (Figure S2A), probably due to spherical aberration, coma and, astigmatism in the microscope. This offset was very small (<10 nm) and does not affect overall resolution (see Figures S2B, C). For the one-dimensional mathematical calculation of localization precision, the mean number of background photons was used as the noise term.

## Supporting Information

Figure S1 Raw data to determine activation efficiency. Red fluorescence of fixed E. coli expressing EGFP immobilized on a cover slip was observed with 532 or 560 nm excitation with regular 488 nm activation pulses (10 ms in every 2 imaging frames) and photon increase in the time series was measured for each buffer composition. The raw image (A, upper) was thresholded to measure only pixels containing bright E. coli (A, lower, pixels labeled with green color was chosen). Iterative adaptive thresholding was used to make a consistency among images. Initial intensity was converted into number of photons by camera calibration data and shown in B (no activation pulse is shown). Inset in B shows the magnified initial part of K3[Fe(CN)6] which showed a tiny increase in red fluorescence. All excitations used 532 nm except K3[Fe(CN)6] which used 560 nm excitation. The sum of photons over time was divided by activation illumination power (W  =  J/s) multiplied by total exposure time (s) to get photon/J in Figure 1B and C. Abbreviations: RiMOS (riboflavin, methionine and oxygen scav-enger), RiM (riboflavin, methionine), OS2 (protocatechuic acid (PCA) + protocatechuate-3,4-dioxgenase (PCD)), OS (oxygen scavenger consisting from glucose oxidase, catalase and glu-cose). (C) Photoconversion efficiency with and without 488 nm activation. Excitation with 532 nm (10 ms exposure at 33 Hz) alone allowed photoconversion of EGFP in RiMOS at low effi-ciency. Abbreviations are the same as in B. PBS (phosphate buffered saline).
(0.22 MB TIF)

Figure S2 Image pre-processing on noisy PALM images. (A) One-dimensional localization error distribution without noise in our simulation (see Figure 2). (B, C) One-dimensional localization error distribution in X (B) and Y (C) direction in de-noised-deconvolved images with different levels of noise. (D) Total processing time of 60 pixels (X) x 60 pixels (Y) x 10,000 time frames on 3.0 GHz eight-core machine. Only denoising and PALM program use parallel processing. Although deconvolution is the time limiting process in our laboratory, commercial deconvolution software (Applied Precision) may have parallel proc-essing capability and in that case total processing time would be much faster. Denoising may take 2–5 times longer if different adaptability and dimension parameters are used.
(0.31 MB TIF)

Figure S3 Comparison of PALM reconstruction in the region of chromosomes and non-chromosome. (A) Denoised wide-field image of chromosomes. DAPI staining is shown in purple and H2AvD-EGFP is in green. (B) PALM reconstruction of chromosome arms boxed in left side of (A). (C) PALM reconstruction of non-chromosome region boxed in the right side of (A). Red arrowhead shows typical filamentous structures characteristic of chromosomes. Bar at the left bottom is 2 µm for (A) and 0.5 µm for (B and C). Note that both images show structures with the cross-sectional diameter of 70–100 nm, but look very different. Thus characteristic structures in chromosomes were unlikely due to reconstruction artifacts.
(2.39 MB TIF)

Figure S4 PALM reconstruction of prometa phase chromosomes in PBS supplemented with Ca2+ and Mg2+. Red arrowheads show typical filamentous structures, and green arrowheads show clefts (hollows) devoid of H2AvD-EGFP. These structures were observed irrespective of fixation buffers used (Figure 3, S3). Inset shows DAPI staining image. Bar at the left bottom is 0.5 µm (3 µm for the inset).
(1.83 MB TIF)

Figure S5 PALM reconstruction of data shown in Figure 3 without denoising and deconvolution. The raw data were processed and presented as in Figure 3 except denoising and deconvolution. The positions of arrows are the same as in Figure 3.
(2.60 MB TIF)

Figure S6 Selection of right Gaussian width removed noise and improve PALM resolution. (A) A histogram of Gaussian width in X and Y directions measured during PALM reconstruction of denoised and deconvolved simulation image with SNR 12.43 (Figure 2). Note that width dis-tribution is very wide with a significant peak around 1.2 pixel. This suggests that peaks smaller than ∼1.0 pixel or larger than ∼1.6 pixel are false positives due to noise. (B) Distribution of Gaussian width in areas of interest (green) and background (red). Inset shows the corresponding green and red-boxed regions of final PALM reconstruction image. This shows that only major peaks are the right PSF sizes and smaller or larger PSFs are actually false positives. The green shade region on the histogram shows the peak width automatically selected by reconstruction program and the rest is thrown away. This approach easily identifies the right PSF shape in any data set, and effectively throw away false positives mostly due to noise. (C) One-dimensional localization precision (red) in the simulated PALM reconstruction of denoised and deconvolved series. Dotted line is before width selection and filled line is after selection. The blue lines show fraction of peaks which constitute the helix among the total number of peaks. Due to false posi-tives and structures in the background images, fraction of correct peaks may not be 100%, but this ratio can be improved by removing peaks with wrong shapes. Note that the image of SNR 7.13 contains anaphase chromosomes (Figure 2C) and its dimension is relatively large (96x106 pixels). Therefore, the fraction of correct peak in this image was lower than other simulation im-ages which are simpler and smaller (60×60 pixels). However, the size of the background is not so relevant to this analysis. The point here is the improvement in excluding false positives, but not comparison among different SNRs.
(0.45 MB TIF)
