# Supplementary figures and images for: Condensed Mitotic Chromosome Structure at Nanometer Resolution Using PALM and EGFP- Histones
Source: PLoS One. 2010 Sep 15;5(9):e12768. doi: 10.1371/journal.pone.0012768 (PMC2939896; doi:10.1371/journal.pone.0012768)

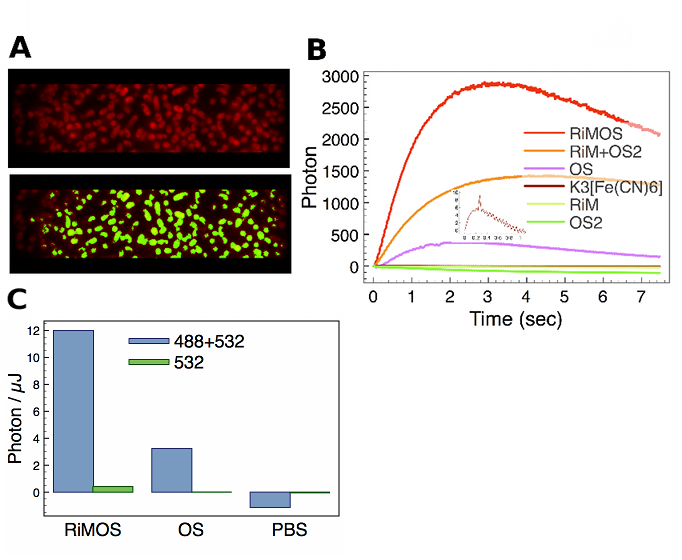

Supplement: Figure S1 — Raw data to determine activation efficiency. Red fluorescence of fixed E. coli expressing EGFP immobilized on a cover slip was observed with 532 or 560 nm excitation with regular 488 nm activation pulses (10 ms in every 2 imaging frames) and photon increase in the time series was measured for each buffer composition. The raw image (A, upper) was thresholded to measure only pixels containing bright E. coli (A, lower, pixels labeled with green color was chosen). Iterative adaptive thresholding was used to make a consistency among images. Initial intensity was converted into number of photons by camera calibration data and shown in B (no activation pulse is shown). Inset in B shows the magnified initial part of K3[Fe(CN)6] which showed a tiny increase in red fluorescence. All excitations used 532 nm except K3[Fe(CN)6] which used 560 nm excitation. The sum of photons over time was divided by activation illumination power (W = J/s) multiplied by total exposure time (s) to get photon/J in Figure 1B and C. Abbreviations: RiMOS (riboflavin, methionine and oxygen scav-enger), RiM (riboflavin, methionine), OS2 (protocatechuic acid (PCA) + protocatechuate-3,4-dioxgenase (PCD)), OS (oxygen scavenger consisting from glucose oxidase, catalase and glu-cose). (C) Photoconversion efficiency with and without 488 nm activation. Excitation with 532 nm (10 ms exposure at 33 Hz) alone allowed photoconversion of EGFP in RiMOS at low effi-ciency. Abbreviations are the same as in B. PBS (phosphate buffered saline). (0.22 MB TIF) [file pone.0012768.s001.tif]

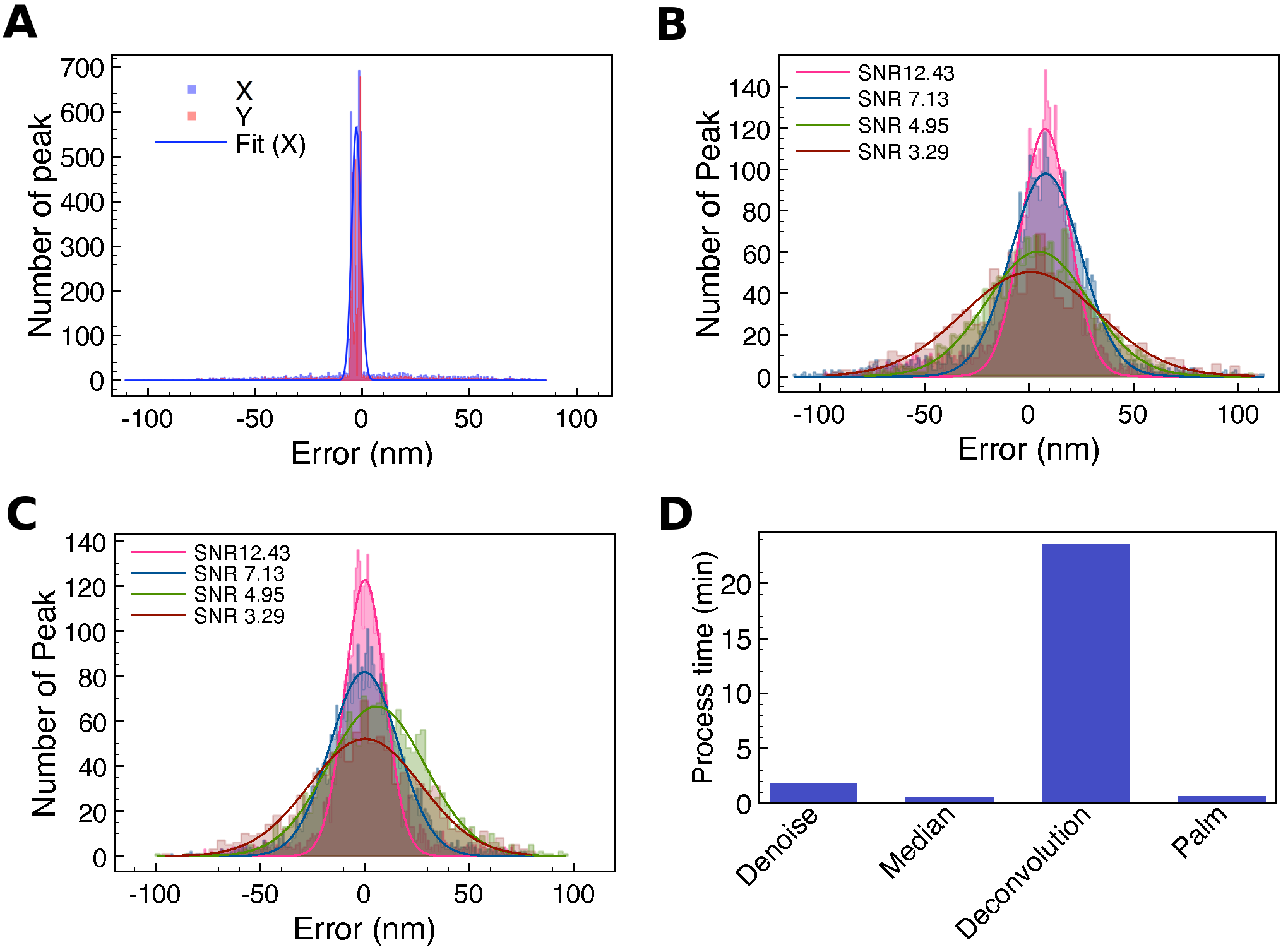

Supplement: Figure S2 — Image pre-processing on noisy PALM images. (A) One-dimensional localization error distribution without noise in our simulation (see Figure 2). (B, C) One-dimensional localization error distribution in X (B) and Y (C) direction in de-noised-deconvolved images with different levels of noise. (D) Total processing time of 60 pixels (X) x 60 pixels (Y) x 10,000 time frames on 3.0 GHz eight-core machine. Only denoising and PALM program use parallel processing. Although deconvolution is the time limiting process in our laboratory, commercial deconvolution software (Applied Precision) may have parallel proc-essing capability and in that case total processing time would be much faster. Denoising may take 2–5 times longer if different adaptability and dimension parameters are used. (0.31 MB TIF) [file pone.0012768.s002.tif]

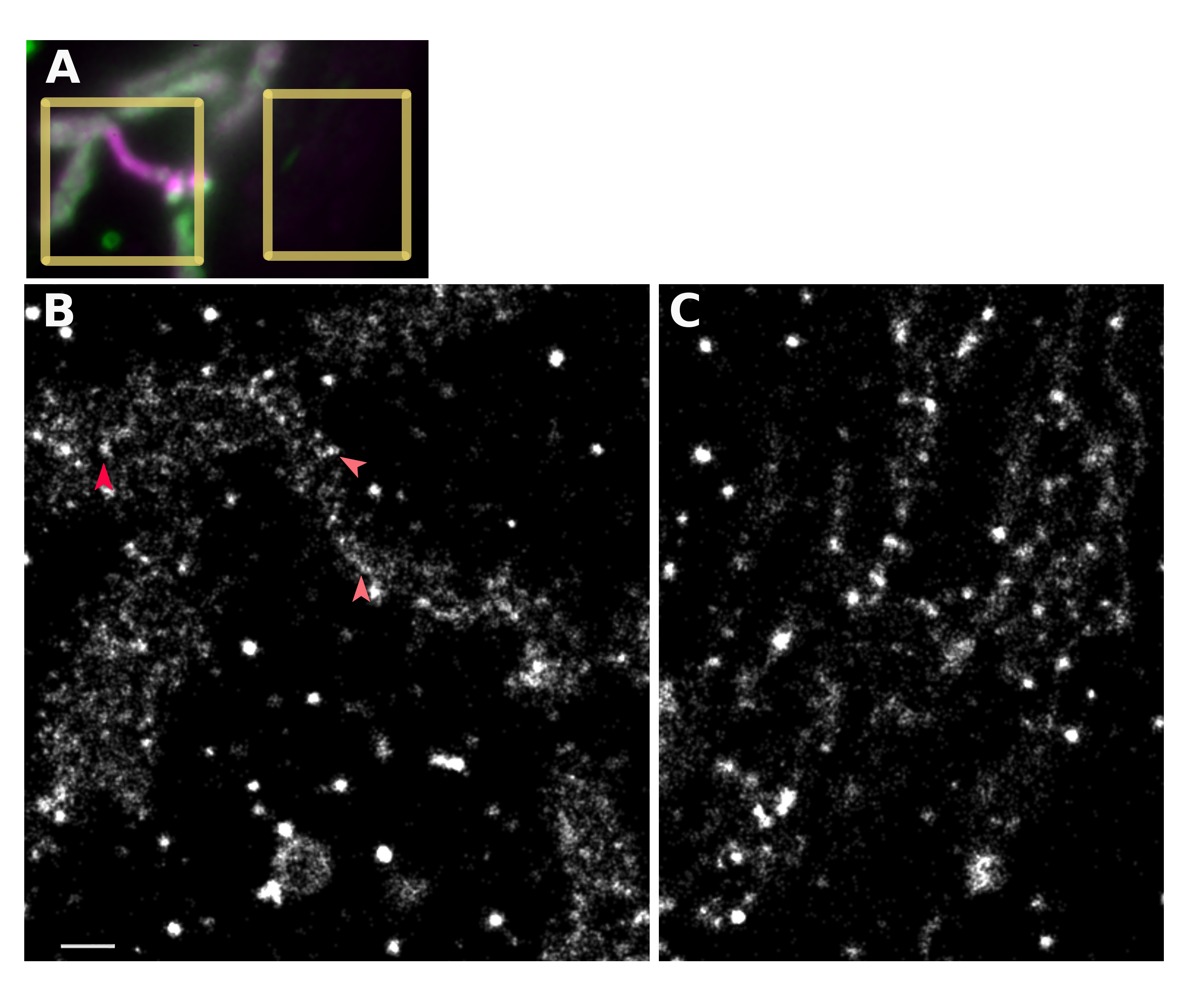

Supplement: Figure S3 — Comparison of PALM reconstruction in the region of chromosomes and non-chromosome. (A) Denoised wide-field image of chromosomes. DAPI staining is shown in purple and H2AvD-EGFP is in green. (B) PALM reconstruction of chromosome arms boxed in left side of (A). (C) PALM reconstruction of non-chromosome region boxed in the right side of (A). Red arrowhead shows typical filamentous structures characteristic of chromosomes. Bar at the left bottom is 2 µm for (A) and 0.5 µm for (B and C). Note that both images show structures with the cross-sectional diameter of 70–100 nm, but look very different. Thus characteristic structures in chromosomes were unlikely due to reconstruction artifacts. (2.39 MB TIF) [file pone.0012768.s003.tif]

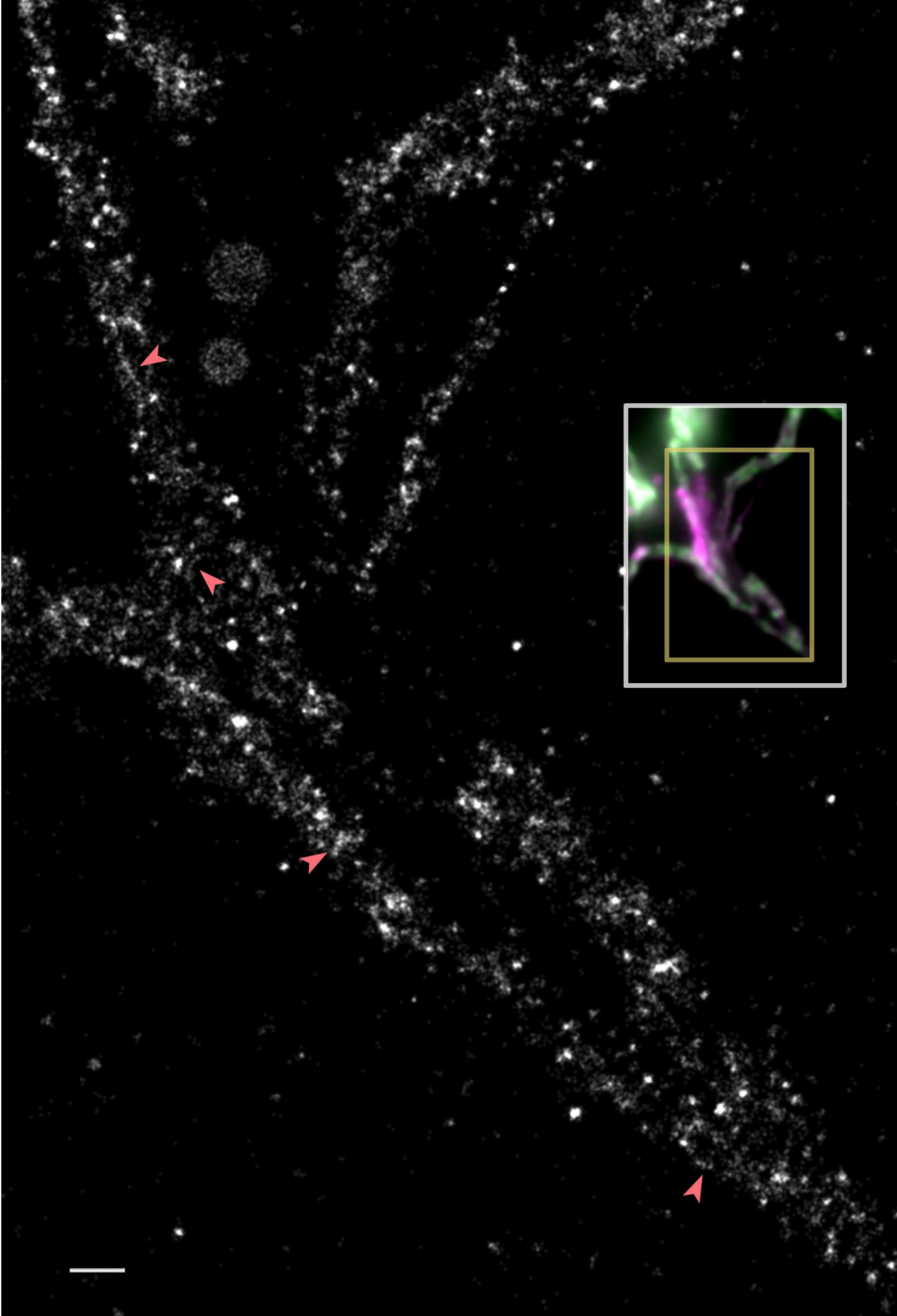

Supplement: Figure S4 — PALM reconstruction of prometa phase chromosomes in PBS supplemented with Ca2+ and Mg2+. Red arrowheads show typical filamentous structures, and green arrowheads show clefts (hollows) devoid of H2AvD-EGFP. These structures were observed irrespective of fixation buffers used (Figure 3, S3). Inset shows DAPI staining image. Bar at the left bottom is 0.5 µm (3 µm for the inset). (1.83 MB TIF) [file pone.0012768.s004.tif]

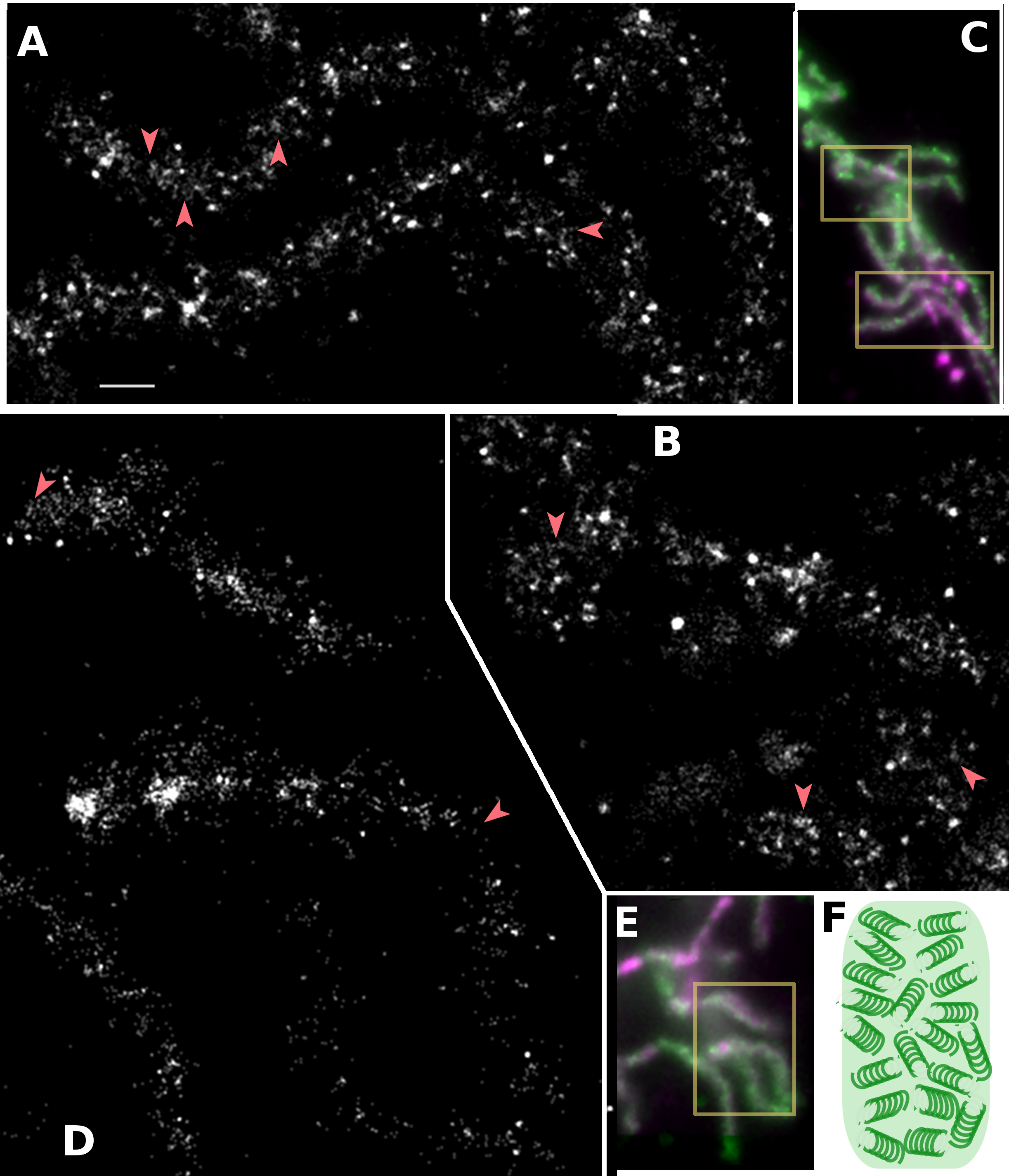

Supplement: Figure S5 — PALM reconstruction of data shown in Figure 3 without denoising and deconvolution. The raw data were processed and presented as in Figure 3 except denoising and deconvolution. The positions of arrows are the same as in Figure 3. (2.60 MB TIF) [file pone.0012768.s005.tif]

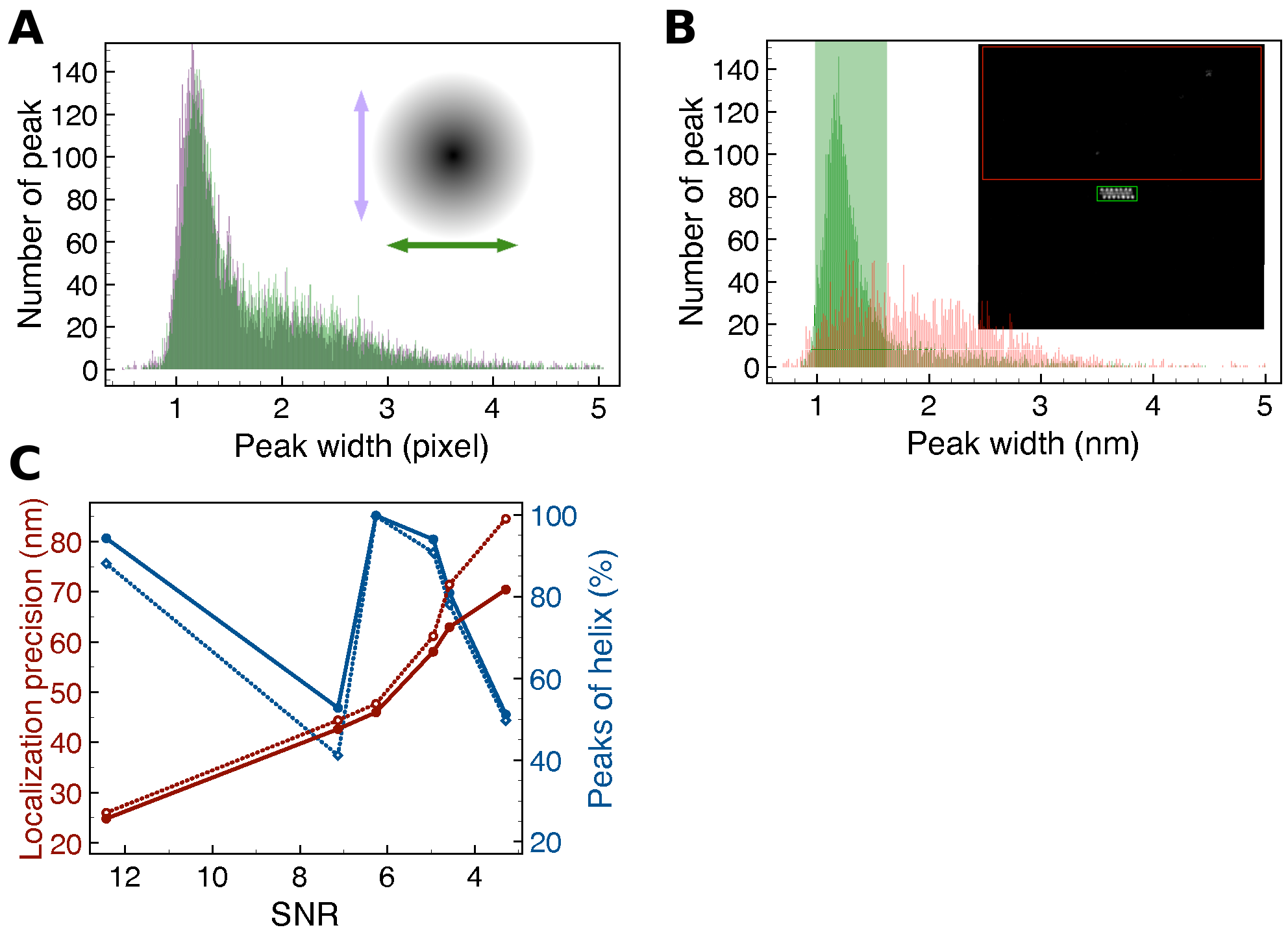

Supplement: Figure S6 — Selection of right Gaussian width removed noise and improve PALM resolution. (A) A histogram of Gaussian width in X and Y directions measured during PALM reconstruction of denoised and deconvolved simulation image with SNR 12.43 (Figure 2). Note that width dis-tribution is very wide with a significant peak around 1.2 pixel. This suggests that peaks smaller than ∼1.0 pixel or larger than ∼1.6 pixel are false positives due to noise. (B) Distribution of Gaussian width in areas of interest (green) and background (red). Inset shows the corresponding green and red-boxed regions of final PALM reconstruction image. This shows that only major peaks are the right PSF sizes and smaller or larger PSFs are actually false positives. The green shade region on the histogram shows the peak width automatically selected by reconstruction program and the rest is thrown away. This approach easily identifies the right PSF shape in any data set, and effectively throw away false positives mostly due to noise. (C) One-dimensional localization precision (red) in the simulated PALM reconstruction of denoised and deconvolved series. Dotted line is before width selection and filled line is after selection. The blue lines show fraction of peaks which constitute the helix among the total number of peaks. Due to false posi-tives and structures in the background images, fraction of correct peaks may not be 100%, but this ratio can be improved by removing peaks with wrong shapes. Note that the image of SNR 7.13 contains anaphase chromosomes (Figure 2C) and its dimension is relatively large (96x106 pixels). Therefore, the fraction of correct peak in this image was lower than other simulation im-ages which are simpler and smaller (60×60 pixels). However, the size of the background is not so relevant to this analysis. The point here is the improvement in excluding false positives, but not comparison among different SNRs. (0.45 MB TIF) [file pone.0012768.s006.tif]
